# Bibliometric analysis of evolutionary trends and hotspots of super-enhancers in cancer

**DOI:** 10.3389/fphar.2023.1192855

**Published:** 2023-07-27

**Authors:** Zhen-Chu Tang, Qiang Qu, Xin-Qi Teng, Hai-Hui Zhuang, Wei-Xin Xu, Jian Qu

**Affiliations:** ^1^ Department of Neurology, The Second Xiangya Hospital of Central South University, Changsha, China; ^2^ Hunan Key Laboratory of Tumor Models and Individualized Medicine, The Second Xiangya Hospital of Central South University, Changsha, China; ^3^ Department of Pharmacy, Xiangya Hospital, Central South University, Changsha, China; ^4^ National Clinical Research Center for Geriatric Disorders, Xiangya Hospital, Central South University, Changsha, China; ^5^ Institute of Hospital Management, Central South University, Changsha, China; ^6^ Hunan Key Laboratory of the Research and Development of Novel Pharmaceutical Preparations, Changsha Medical University, Changsha, China; ^7^ Department of Pharmacy, The Second Xiangya Hospital, Institute of Clinical Pharmacy, Central South University, Changsha, China

**Keywords:** super-enhancer, bibliometric analysis, cancer, hot spots, trends

## Abstract

**Introduction:** In the past decade, super-enhancer (SE) has become a research hotspot with increasing attention on cancer occurrence, development, and prognosis. To illustrate the hotspots of SE in cancer research and its evolutionary tendency, bibliometric analysis was carried out for this topic.

**Methods:** Literature published before Dec 31, 2022, in WOSCC, was systematically classified, and Citespace, bibliometric.com/app, and GraphPad Prism analyzed the data.

**Results:** After screening out inappropriate documents and duplicate data, 911 publications were selected for further bibliometric analysis. The top five research areas were Oncology (257, 28.211%), Cell Biology (210, 23.052%), Biochemistry Molecular Biology (209, 22.942%), Science Technology Other Topics (138, 15.148%), and Genetics Heredity (132, 14.490%). The United States of America (United States) has the highest number of documents (462, 50.71%), followed by China (303, 33.26%). Among the most productive institutions, four of which are from the United States and one from Singapore, the National University of Singapore. Harvard Medical School (7.68%) has the highest percentage of articles. Young, Richard A, with 32 publications, ranks first in the number of articles. The top three authors came from Whitehead Institute for Biomedical Research as a research team. More than two-thirds of the research are supported by the National Institutes of Health of the United States (337, 37.654%) and the United States Department of Health Human Services (337, 37.654%). And “super enhancer” (525), “cell identity” (258), “expression” (223), “cancer” (205), and “transcription factor” (193) account for the top 5 occurrence keywords.

**Discussion:** Since 2013, SE and cancer related publications have shown a rapid growth trend. The United States continues to play a leading role in this field, as the top literature numbers, affiliations, funding agencies, and authors were all from the United States, followed by China and European countries. A high degree of active cooperation is evident among a multitude of countries. The role of SEs in cell identity, gene transcription, expression, and inhibition, as well as the relationship between SEs and TFs, and the selective inhibition of SEs, have received much attention, suggesting that they are hot issues for research.

## 1 Introduction

Regulation of gene expression relies on the cis-regulatory elements in cells, which effectively regulate their target genes and accurately assemble gene expression programs by binding to transcription factors (TFs). Enhancer is a type of DNA cis-regulatory element that combines with TFs to promote gene expression (Hnisz et al., 2013; Shang et al., 2019). Super-enhancer (SE) is another type of regulatory element identified as a large group of enhancers with an average span of more than 20 kb (Hnisz et al., 2013). Chen and colleagues first proposed the term “super-enhancer” in 2004 to define a functional enhancer of the homologo-3 of silkworm nuclear polyhedrosis virus (Shang et al., 2019). In recent years, Young and colleagues have vigorously promoted the concept of SE and determined its critical role in controlling cell identity and disease (Hnisz et al., 2013; Sabari and Dall'Agnese, 2018). Compared to the typical enhancer, SE has a higher level of activity enhancers histone modification, such as H3K27ac and H3K4me1, and enriches higher density master TFs and cofactors, such as the mediator complex (such as MED1), bromodomain-containing proteins (such as BRD4), cycle-dependent kinase 7(CDK7) and p300 (Meng et al., 2018). In addition, RNA pol II is highly enriched in SE (Shin, 2018; Zheng et al., 2020). Therefore, SE drives stronger transcriptional activity than typical enhancers (Whyte et al., 2013; He et al., 2019). Meanwhile, super enhancer-related genes are particularly sensitive to RNAP Ⅱ mediated transcription and small disturbance of CDK7 kinase function (Kwiatkowski et al., 2014). The identification of enhancers and SEs is often based on Chip-seq of enhancer-associated TFs and their cofactors (MED1, BRD4) or histone modifications (H3K27ac, H3K4me1) (Meng et al., 2018).

The SE model has generated much interest in the past decade, as it is responsible for dysfunctional or disease states. It has been demonstrated that SEs regulate the transcription of cell-type-specific genes, determine the identity and fate of cells (Fox et al., 2020), and play critical regulatory roles in cell growth, differentiation, and disease development (Aspuria et al., 2020; Sun et al., 2020). It has also been shown to be related to the initiation and development of various diseases, including certain autoimmune diseases (Peeters et al., 2015; Vahedi et al., 2015), diabetes (Sun et al., 2018), neurodegenerative diseases (Hnisz et al., 2013), and various types of tumors (Dong et al., 2021; Gartlgruber et al., 2021; Sang et al., 2022).

Genetic and epigenetic changes drive cancer-related gene transcription disorders, which play a crucial role in the occurrence and development of cancer (Sengupta and George, 2017). SE has been reported to activate the overexpression of key oncogenes in various tumors. Tumor cells can form carcinogenic SEs in key oncogenes through mutation, epigenetic change, or chromosome remodeling, resulting in dysregulated transcriptional regulatory components binding, thus promoting carcinogenic transcription and tumor progression (Bradner et al., 2017). A type of non-coding RNAs transcribed from DNA sequences located in the super-enhancer regions is referred to as “super-enhancer RNAs (seRNAs).” (Peng et al., 2019; Tan et al., 2020). seRNAs regulate multiple tumors’ malignant progression (Peng et al., 2019; Tan et al., 2020). Moreover, seRNAs promote transcription by enhancing the formation of SE-gene promoter loops (Wu and Shen, 2019; Xiang-Ping Li et al., 2023). Diverse TFs and coactivators (such as BRD4 and MED1) may enrich the SEs and form phase-separated condensates to drive oncogene expression (Sabari and Dall'Agnese, 2018; Boija et al., 2018). Nowadays, more and more studies show that SEs play critical regulatory roles in multiple biological functions of cancers, including tumor cell proliferation and migration (Wang et al., 2021), cell cycle (Nakamura et al., 2017), immune response (Yu et al., 2021), and chemo-sensitivity (Li et al., 2021). Since tumor-related variants are significantly enriched in SE, therapies targeting SEs may become new strategies for cancer treatment (Zhang et al., 2020). Moreover, the expression of SE-induced tumor-associated genes may contribute to the is related to diagnosis and prognosis of cancer and cancer precision medicine (Ren et al., 2022).

With the improving awareness of the key role of SEs in cancer malignant progression and the advanced epigenetic technology, SE and cancers related research has mushroomed and increased rapidly. Bibliometric analysis has been used to analyze research frontiers and progress chronologically (Chen and Song, 2019). This study used bibliometric software, including CiteSpace and Vosviewer, to analyze relevant data. We aim to provide a comprehensive overview of the current research trends in the relationship between SE and cancer, aiming to facilitate a deeper understanding of this field.

## 2 Methods

### 2.1 Data collection

**FIGURE 1:**
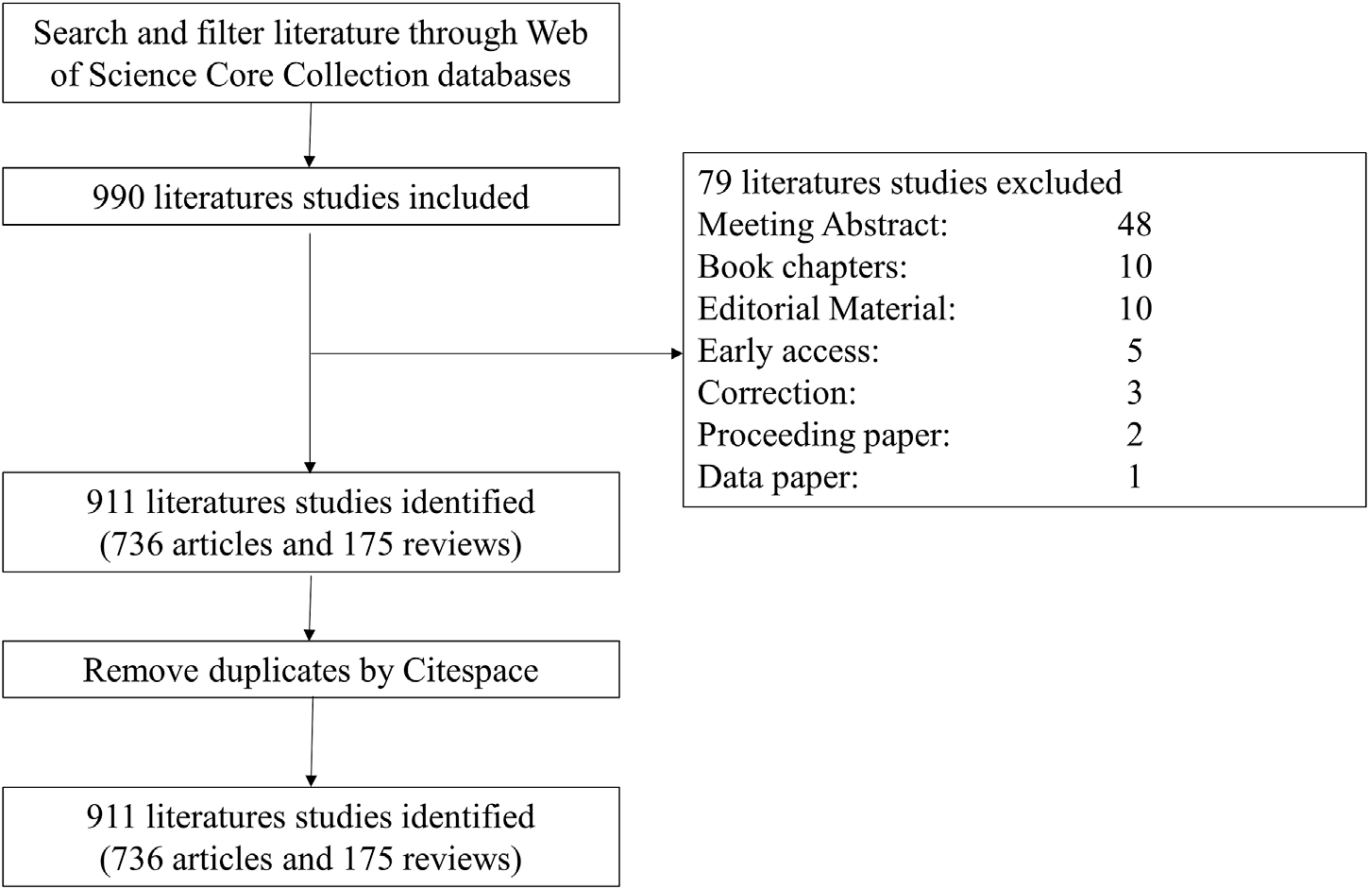
Diagram of the literature screening process.

**Literature** published before Dec. 31, 2022, was identified by searching the Web of Science Core Collection (WoSCC) with the following strategy: [(TS= (cancer* OR tumor* OR neoplasm* OR Neoplasia*)] AND TS= [(“super enhancer*” OR “super-enhancer*”)] AND LA= (English), which yielded 990 documents. We only selected articles and reviews and eliminated other types of documents, obtaining 911 documents, including 736 articles and 175 reviews. (The selection procedure is depicted in Figure 1).

### 2.2 Data analysis

We used CiteSpace software (version 6.2. R3) and bibliometric.com/app to analyze the retrieved literature data, including SE and cancer-related publications’ institutions, authors and their countries, keywords of publications, and other indicators were extracted to evaluate the quality of the publications. And we used the WoSCC to analyze the associated data.

## 3 Results

### 3.1 General information and publication date

**FIGURE 2:**
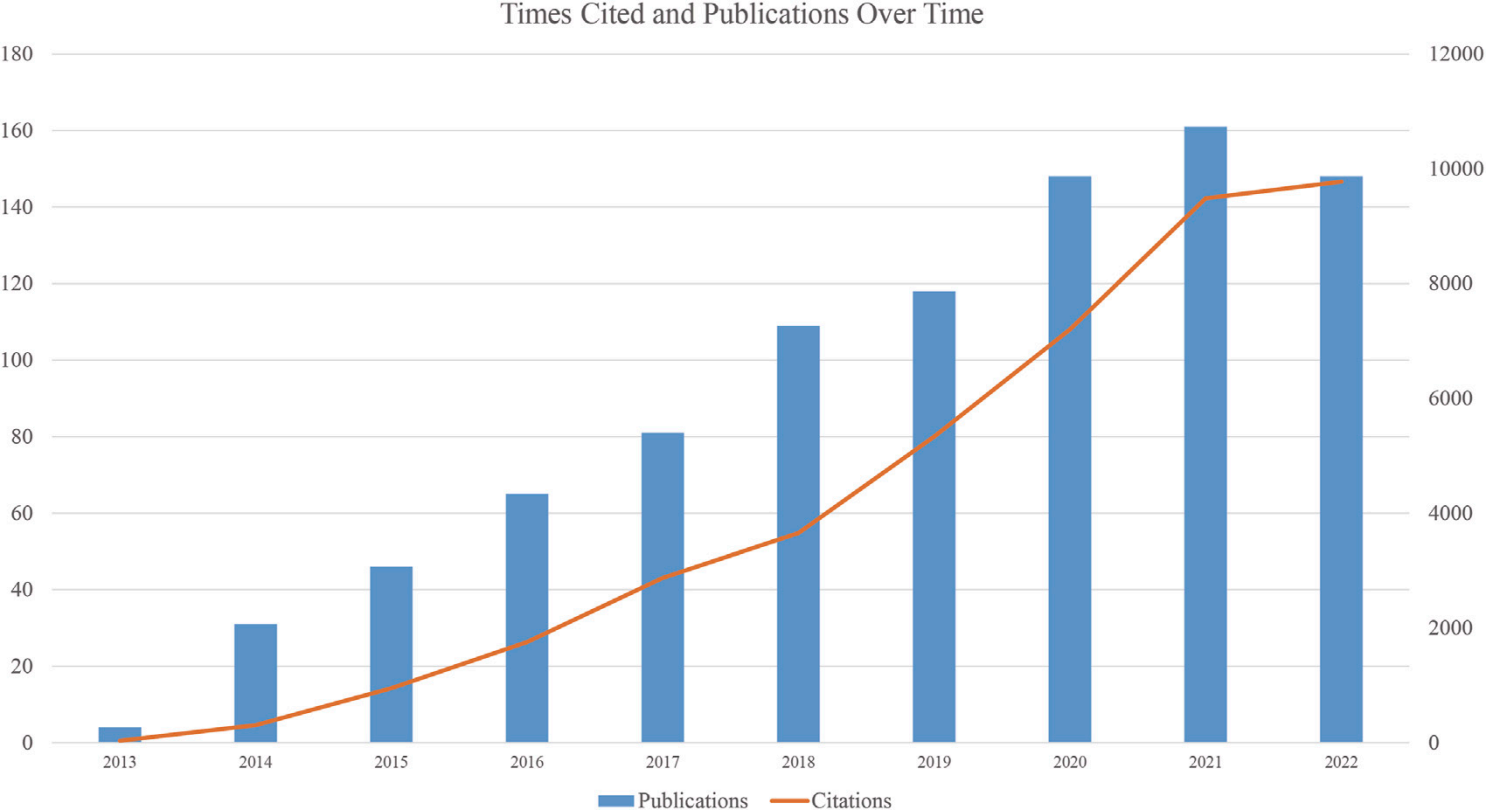
Publications and Citations over time.

We collected 911 publications, and 736 articles and 175 reviews were included. After removing self-citations, there were 26, 298 cited articles with 39, 017 citation frequencies. The average number of citations per article is 49.34, and the h-index was 102. Figure 2 shows the annual number of publications. The publication of relevant articles started with four articles in 2013, and the trend has been rising for years. As of 31 December 2022, 148 related papers have been published in 2022. The top five research areas were Oncology (257, 28.211%), Cell Biology (210, 23.052%), Biochemistry Molecular Biology (209, 22.942%), Science Technology Other Topics (138, 15.148%), and Genetics Heredity (132, 14.490%). In this field, the most published journals are NATURE COMMUNICATIONS (63, 6.915%), NUCLEIC ACIDS RESEARCH (30, 3.293%), CELL REPORTS (27, 2.964%), CANCER RESEARCH (23, 2.525%), CELL (19, 2.086%), NATURE GENETICS (19, 2.086%) (Table 1).

**TABLE 1:** Top five research areas, published journals, and funding agencies based on the number of documents (2013–2022).

Field	Top five	Record count (%)
Research Areas	Oncology	257 (28.211%)
	Cell Biology	210 (23.052%)
	Biochemistry Molecular Biology	209 (22.942%)
	Science Technology Other Topics	138 (15.148%)
	Genetics Heredity	132 (14.490%)
Journals	*Nature Communications*	63 (6.915%)
	*Nucleic Acids Research*	30 (3.293%)
	*Cell Reports*	27 (2.964%)
	*Cancer Research*	23 (2.535%)
	*Cell*	19 (2.086%)
	*Nature Genetics*	19 (2.123%)
Funding Agencies	the National Institutes of Health (NIH United States)	309 (33.919%)
	United States Department of Health Human Services	309 (33.919%)
	National Natural Science Foundation Of China	202 (22.173%)
	Nih National Cancer Institute	130 (14.270%)
	Ministry Of Education Culture Sports Science And Technology	48 (5.269%)

### 3.2 Distributions of countries and institutions

**FIGURE 3:**
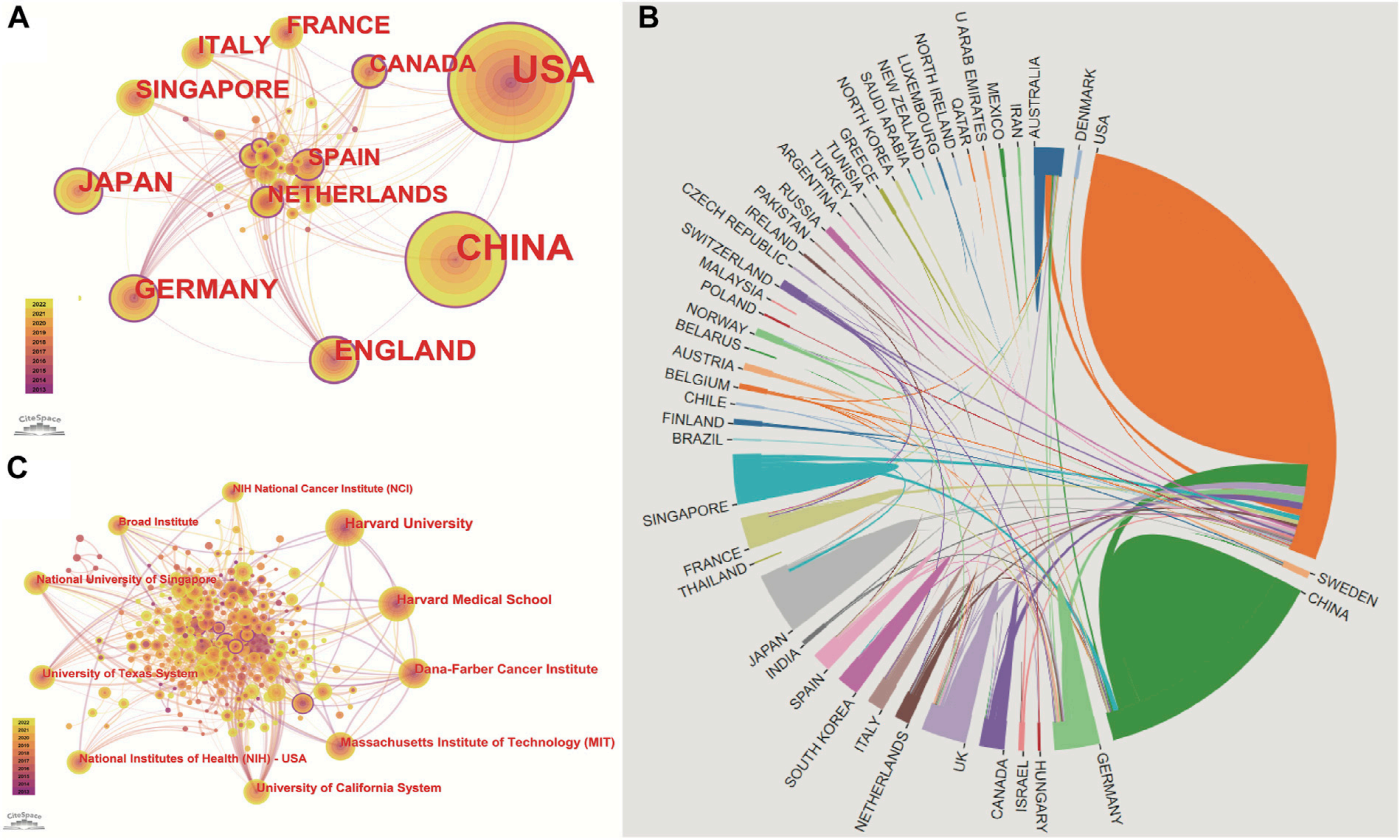
Publications over countries/institutions. The figure shows the top countries regarding the number of published articles. The United States ranks first for the most productive countries, followed by China, Germany, England, Japan, Singapore, Italy, France, and the Netherlands. **(A)** node’s size indicates how many articles are published by countries/institutions. Cooperation between countries/institutions is represented by the line between each node. The nodes with a purple outer circle represent their centrality higher than 0.1. **(B)** shows that China and the United States are high-yield countries, and about 1/5 of the articles in the United States are produced through cooperation between countries. In contrast, the number of cooperative articles in China is less than 10%. **(C)** Institutions co-occurrence map of publications. The size of the node represents the number of publications. The lines between the nodes represent the cooperation between the different institutions.

These articles were from 48 countries/regions and 1,448 institutions. The most literature contributing countries/regions is the United States (462, 50.71%), followed by China (303, 33.26%), England (75, 8.233%), Germany (72, 7.903%), and Japan (71, 7.794%). The contributions and cooperation between countries are visualized in Figures 3A,B. Active cooperation can be seen among countries. The number of articles can be displayed from the node size. Figure 3C and Table 2 showed that four of the top five institutions as for article count are from the United States, namely, Harvard Medical School (70, 7.68%), DANA Farber Cancer Institution (67, 7.35%), Massachusetts Institution of Technology (44, 4.83%), NIH National Cancer Institute (36, 3.95%). And one is from Singapore, the National University of Singapore (46, 5.05%). The major funding agencies include the National Institutes Of Health (309, 33.919%), the United States Department Of Health Human Services (309, 33.919%), National Natural Science Foundation Of China (202, 22.173%), NIH National Cancer Institute (130, 14.270%), Ministry Of Education Culture Sports Science And Technology (48, 5.269%) (Table 1).

**TABLE 2:** Top five countries/regions and institutions based on the number of documents (2013–2022). The data of record count is obtained by setting the K value in g-index to 25 in citespace. H-index is from the website of science’s citation report.

Field		Record count (%)	Centrality	H-index
Countries	United States	462 (51.71%)	0.3	91
	China	303 (33.26%)	0.15	44
	England	75 (8.233%)	0.31	37
	Germany	72 (7.903%)	0.1	31
	Japan	71 (7.794%)	0.11	21
Affiliations	Harvard Medical School	70 (7.68%)	0.11	48
	DANA Farber Cancer Institution	67 (7.35%)	0.13	47
	National University of Singapore	46 (5.05%)	0.07	23
	Massachusetts Institution of Technology	44 (4.83%)	0.08	46
	NIH National Cancer Institute	36 (3.95%)	0.13	20

### 3.3 Contribution of authors and co-cited authors

**FIGURE 4:**
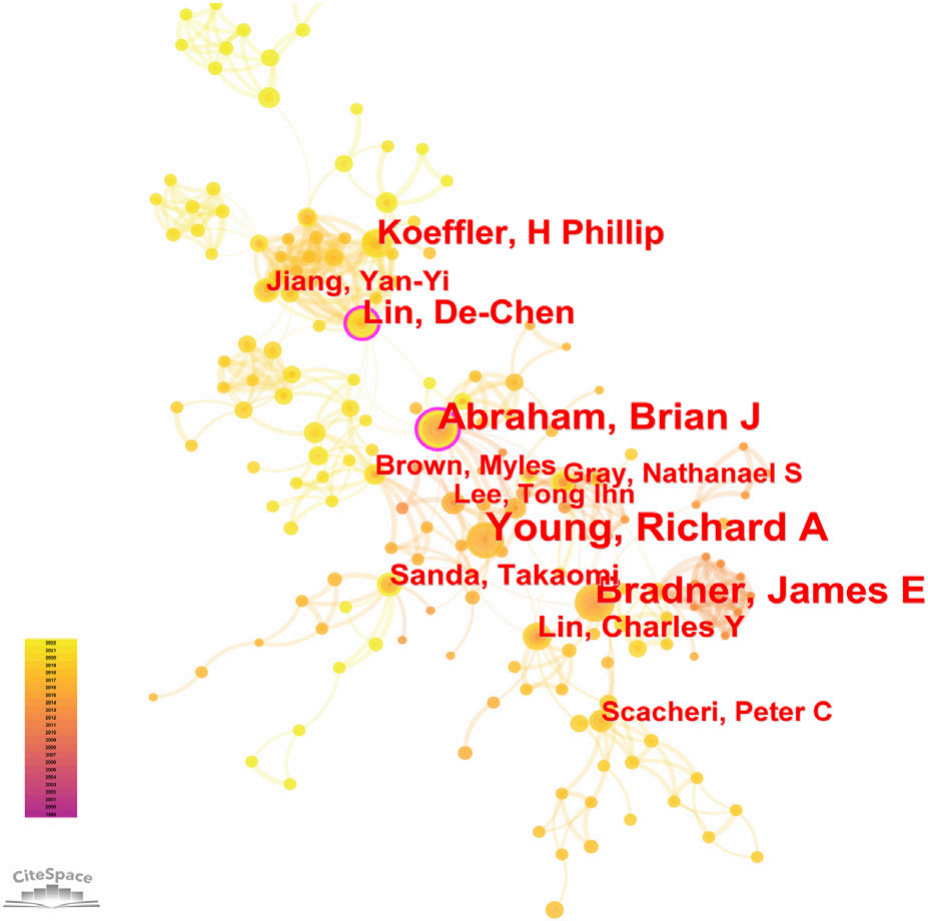
Co-occurrence authors’ network.

**FIGURE 5:**
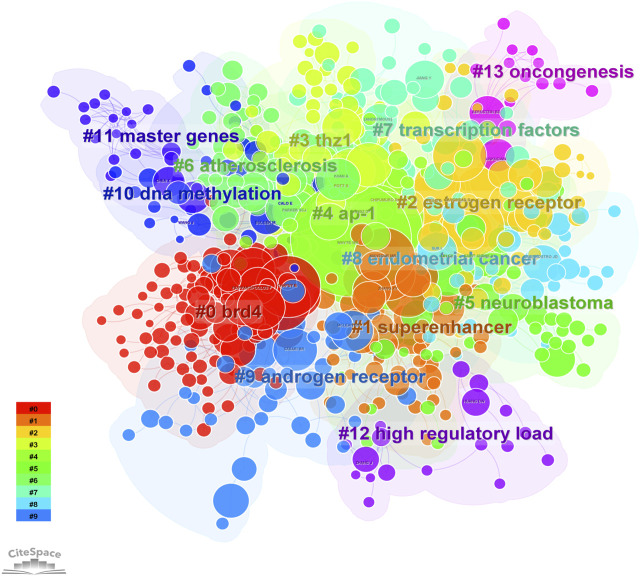
The cluster of cited authors. Keyword cluster analysis (2013–2022). A total of 15 clusters are distinguished by different colors. Cluster #0 is the largest, followed by cluster #1, and so on. A cluster label was assigned by CiteSpace based on the terms that appear most frequently in relevant articles. The top five clusters are “brd4,” “super enhancer,” “estrogen receptor,” “thz1,” and “ap-1”.

8, 268 authors have joined the research field of SE and cancer. Table 3 shows that the five most prolific authors analyzed by bibliometric.com/app are Young, Richard A (Whitehead Institute for Biomedical Research, count 32), Bradner, James E (Whitehead Institute for Biomedical Research, count 23), Abraham, Brian J (Whitehead Institute for Biomedical Research, count 22), Lin, CY (Whitehead Institute for Biomedical Research, count 15), Lin, DC (National University of Singapore, count 15) in rank order. Interestingly, the top four authors with the highest number of publications come from the same institution as a research team. Lin, De-Chen came from National University of Singapore. Authors with high citation rates were Young, Richard A (2,348 citations), Abraham, Brian J (1,391 citations), Lee, TI (1,382 citations), Bradner, James E (1249 citations), and Hoke, HA (1,102 citations). Moreover, Table 3 also shows the first author or corresponding author with the highest number of articles and citations. The co-occurrence authors’ network is shown in Figure 4. Nodes represent co-cited authors; Nodes are sized according to their citations; lines between them indicate how many authors collaborated on the paper. Table 3 shows the top 5 co-cited authors and HNISZ D is the most co-cited author (628 citations). Figure 5 shows the clusters of the cited authors, which can be divided into 15 clusters; the largest one is cluster #0, followed by cluster# 1, and so on. The top five clusters are “brd4,” “super enhancer,” “estrogen receptor,” “thz1,” and “ap-1”.

**TABLE 3:** Top five authors, co-cited authors and the citations.

Authors	Affiliation	Count	First published year	Authors	Total number of citations	Authors	Average citation numbers	Authors	Article numbers by the first author
Young RA	Whitehead Institute for Biomedical Research	32 (3.575%)	2013	Young, RA	2,348	Saint-Andre, V	597	Gryder, BE	4
Bradner JE	Whitehead Institute for Biomedical Research	23 (2.525%)	2013	Abraham, BJ	1,391	Hoke, HA	551	Hamdan, FH	4
Abraham BJ	Whitehead Institute for Biomedical Research	22 (2.415%)	2013	Lee, TI	1,382	Lau, A	551	Li, X	4
Charles Y Lin	Whitehead Institute for Biomedical Research	15 (1.676%)	2013	Bradner, JE	1,249	Sigova, AA	328.5	Hnisz, D	3
Lin, De-Chen	National University of Singapore	15 (1.676%)	2017	Hoke, HA	1,102	Loven, J	263	Wang, X	3

Co-cited Authors	Affiliations	Citation	Centrality	Authors	Citation numbers of first author	Authors	Average citation numbers for the first author	Authors	Article numbers by corresponding authors
HNISZ D	Max Planck Institute for Molecular Genetics	628	0.01	Hnisz, D	763	Loven, J	505	Young, RA	11
WHYTE WA	Whitehead Institute for Biomedical Research	582	0.03	Loven, J	505	Hnisz, D	254.33	Sanda, T	9
LOVEN J	Whitehead Institute for Biomedical Research	504	0.01	Chapuy, B	157	Chapuy, B	157	Fullwood, MJ	6
ZHANG Y	National University of Singapore	234	0.08	Mansour, MR	145	Mansour, MR	145	Vakoc, CR	5
HEINZ S	University of California San Diego	170	0.02	Chipumuro, E	121	Chipumuro, E	121	Jiang, YY	4

### 3.4 Topic and frontiers of SE in cancer research

#### 3.4.1 Top 10 highly cited reference

**FIGURE 6:**
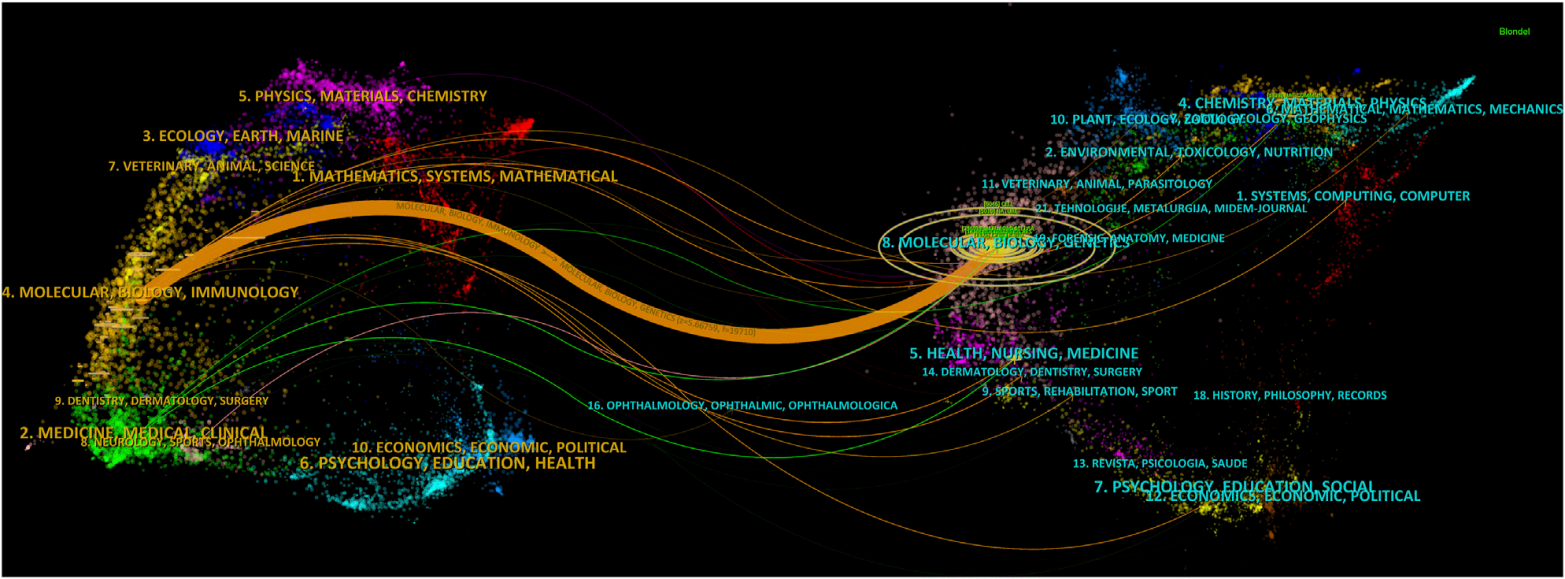
A dual-map overlay of the science mapping literature. There is a colored path representing the citation relationship between cited journals on the right and citing journals on the left. Using citation regions’ colors, we can distinguish the trajectory of citations. These trajectories thicken with increasing z-score frequency.

To reflect the hot spots and depth of research about SEs and cancer, we showed the top 10 highly cited references in Table 4. The top 4 high-cited articles were all published in *Cell* by Young, Richard A and his colleagues. Based on the first top 3 articles, Richard A and his colleagues proposed the SEs concept. Most genes that control pluripotency are enhanced by master transcription factors found in embryonic stem cells. Domains called SEs consist of clusters of enhancers that master regulators and mediators occupy densely. Figure 6 shows the dual-map coverage of the literature. The reference relationship is represented by the color path, and the color of the citing regions represents the citation trajectories. The thickness of these trajectories is proportional to the citation frequency of the z-score. Our dataset mainly contained two citation paths: 4. Molecular, biology, immunology, and 2. Medicine, medical, clinical. The literature was mainly affected by the following domains: 8. Molecular, biology, genetics, and 4. Chemistry, material physics (Figure 6).

**TABLE 4:** Top 10 high-cited references related to the role of super-enhancer in cancer.

Title	Corresponding authors	Journal	Year	Cited counts	Impact factor (2022 years)
Super-enhancers in the control of cell identity and disease	Richard A Young	Cell	2013	220	64.5
Master transcription factors and mediator establish super-enhancers at key cell identity genes	Richard A Young	Cell	2013	220	64.5
Selective inhibition of tumor oncogenes by disruption of super-enhancers	Richard A Young	Cell	2013	210	64.5
Transcriptional Addiction in Cancer	Richard A Young	Cell	2017	84	64.5
Discovery and characterization of super-enhancer-associated dependencies in diffuse large B cell lymphoma	James E Bradner	Cancer Cell	2013	76	50.3
Identification of focally amplified lineage-specific super-enhancers in human epithelial cancers	Matthew Meyerson	Nat Genet	2016	76	30.8
Oncogene regulation. An oncogenic super-enhancer formed through somatic mutation of a noncoding intergenic element	Richard A Young	Science	2014	74	56.9
Convergence of developmental and oncogenic signaling pathways at transcriptional super-enhancers	Richard A Young	Mol Cell	2015	73	16.0
What are super-enhancers?	Jason D Lieb	Nat Genet	2015	73	30.8
Super-Enhancer-Driven Transcriptional Dependencies in Cancer	Rani E George	Trends Cancer	2017	73	18.4

#### 3.4.2 Keyword co-occurrence

**FIGURE 7:**
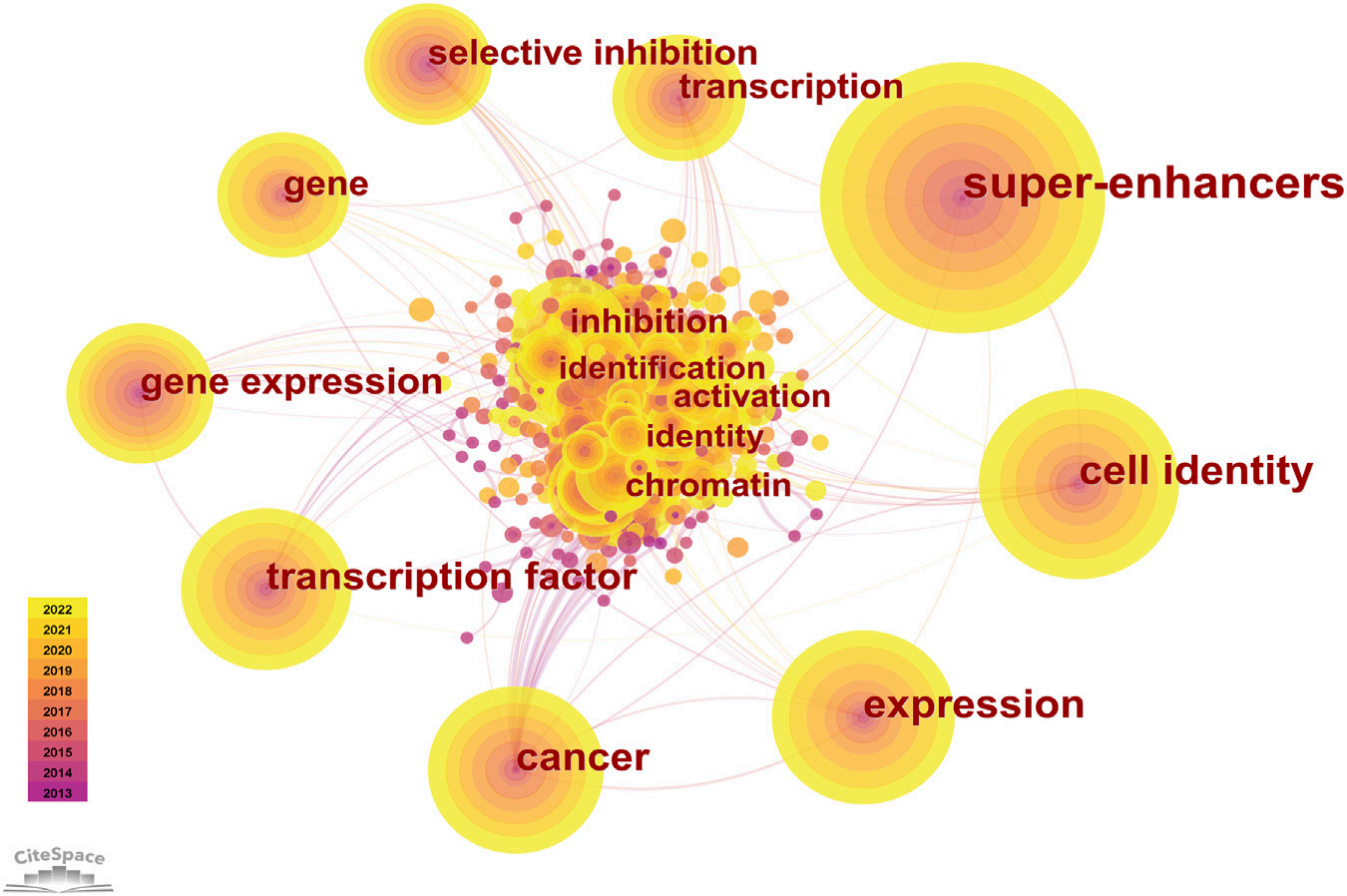
Keywords co-occurrence network. The node size represents the count of keywords. Lines connecting nodes indicate co-occurrence frequencies; the thicker, the higher.

Keyword analysis was conducted. Co-occurrence is the appearance of two or more keywords in a single piece of literature. Based on the analyzed literature, a keyword co-occurrence map is presented. In Table 5, high co-occurrence keywords are listed. And the top 5 keywords are “super enhancer” (525), “cell identity” (258), “expression” (223), “cancer” (205), and “transcription factor” (193). A keyword co-occurrence network map was drawn for a further acquaintance in Figure 7.

**TABLE 5:** Top 25 keyword co-occurrence frequency to the role of super-enhancer in cancer.

Keywords	Count	Centrality	Year
super-enhancers	525	0.01	2014
cell identity	258	0.02	2014
expression	223	0.01	2014
cancer	205	0.03	2013
transcription factor	193	0.01	2014
gene expression	144	0.06	2013
gene	119	0.01	2014
selective inhibition	114	0.01	2014
transcription	112	0.04	2015
inhibition	95	0.02	2015
chromatin	85	0.02	2013
activation	81	0.04	2014
identity	70	0.05	2015
identification	69	0.02	2014
differentiation	67	0.02	2015
c myc	65	0.08	2013
mutation	50	0.02	2014
dna methylation	48	0.1	2013
protein	47	0.01	2014
binding	43	0.02	2015
genome	43	0.06	2014
landscape	42	0.03	2015
proliferation	40	0.01	2017
long noncoding rna	36	0.04	2016
resistance	35	0.02	2016

#### 3.4.3 Keyword burst and cluster timeline

**FIGURE 8:**
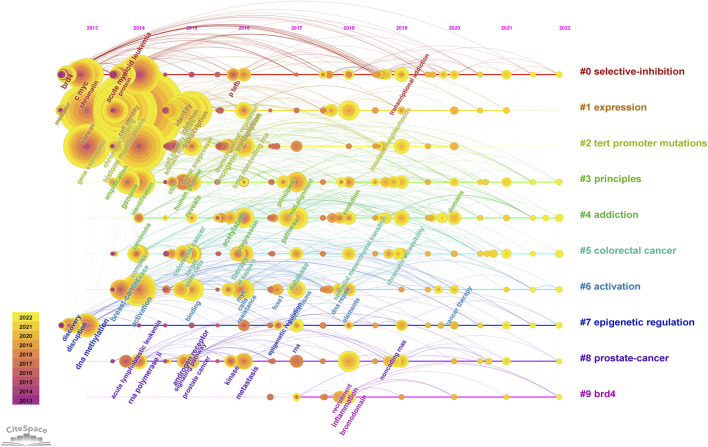
Keyword cluster timeline analysis. An evolution of research hotspots based on time is shown using timeline maps. Time can be determined by the length of a cluster’s horizontal straight line. We showed 10 clusters and color-coding was used to distinguish them. Cluster #0 is the largest cluster, followed by cluster #1, and so on. Using this timeline chart, we can see how keywords emerged according to the time they were included in the cluster.

**FIGURE 9:**
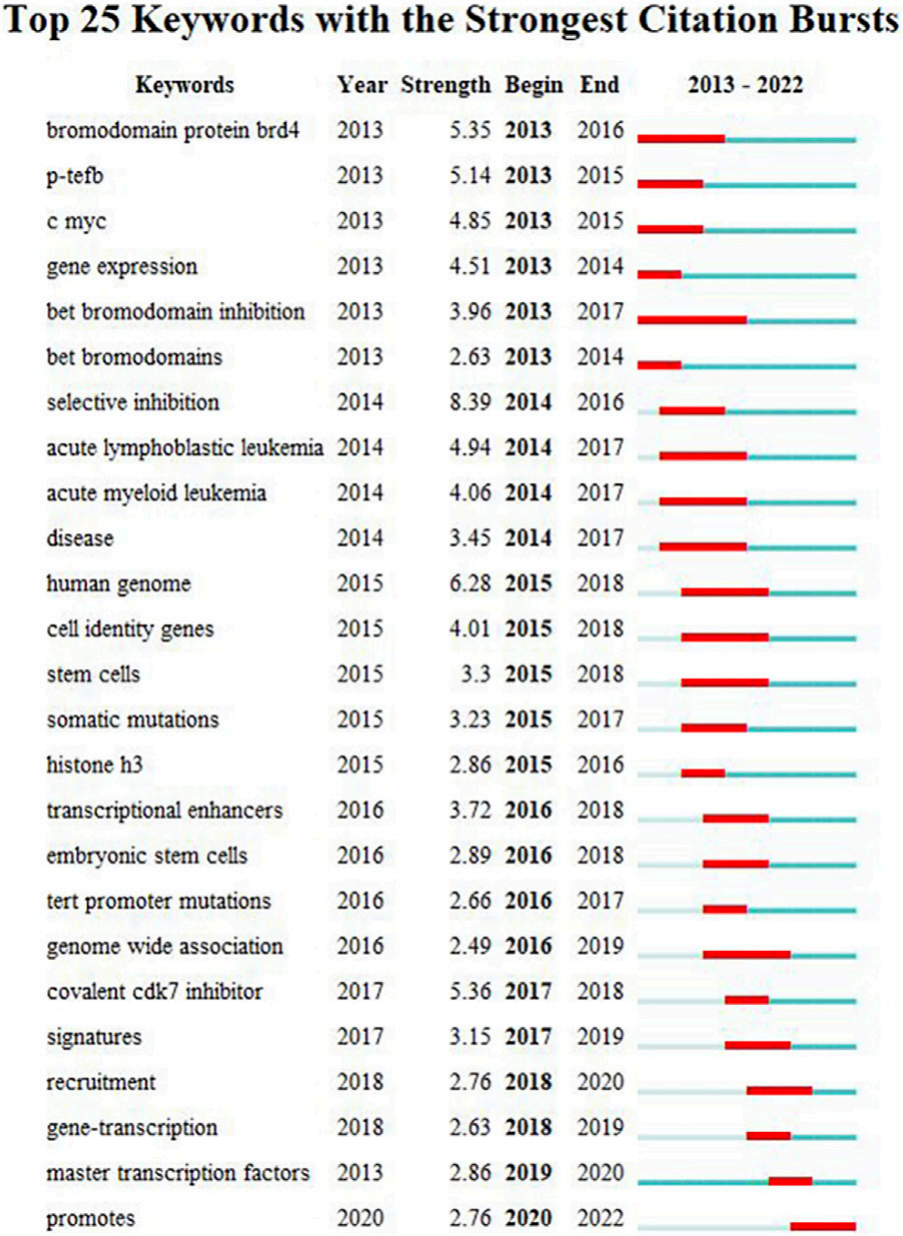
Top 25 keywords with the strongest citation bursts. During a particular period, there was a spike in citations for a particular keyword, which indicates the research Frontier within a specific time. Citation bursts are indicated by blue lines and periods by red lines. Burst intensity is indicated by the number in parentheses. The larger the number, the higher the intensity of the burst.

We used CiteSpace to carry out keyword cluster timeline analysis. Figure 8 shows the keyword timeline. Timeline chart showing keywords in clusters by their appearance date. Keyword color matches cluster label color. A total of ten clusters are generated: “selective-inhibition,” “expression,” “tert promoter mutations,” “principles,” “addiction,” “colorectal cancer,” “activation,” “epigenetic regulation,” “prostate-cancer,” “brd4”. Moreover, Figure 9 also showed the top 25 keywords with the strongest citation bursts. “selective inhibition” (8.39) has the highest strength, followed by “human genome” (6.28), and “covalent cdk7 inhibitor” (5.36). Long-lasting citation bursts occur for keywords such as “bromodomain protein brd4” (2013–2016), “bet bromodomain inhibition” (2013–2017), “acute lymphoblastic leukemia” (2014–2017), “acute myeloid leukemia” (2014–2017), “cell identity gene” (2015–2018), indicating that studies on these directions get more researchers’ attention. “Promotes” (2019–2022) was the latest burst keywords.

## 4 Discussion

### 4.1 Research trends

According to our bibliometric analysis, scientific production in SEs and cancer has been growing since 2013. Among the top 9 journals with the most published articles, the influence factors 8 are above 10 points. These reflect the significance of SEs in cancer research. The five contributors with the largest publication amounts are the United States, China, Germany, England, and Japan. It is worth noting that the top four funding institutions supporting the largest research are also from the United States and China. This shows that scientific research cannot be separated from adequate financial support and leading research institutions. Moreover, cooperation between countries is becoming increasingly frequent and conducive to publishing high-quality articles. Among the top five institutions—Harvard Medical School, Dana Farber Canc Inst, Natl Univ Singapore, Massachusetts Institution of Technology, National Cancer Institute—four belong to the United States and one to Singapore. Among the 8,268 authors who participated in the SEs and cancer research field, the most prolific author is Young, Richard A from the Whitehead Institute for Biomedical Research. And the first five highly cited articles in this field are all from Young, Richard A, and his colleagues, whose research vigorously promoted the concept of SE and determined its critical role in cancer. Thus, in the field of SEs and cancers, the United States is in the leading position in the world regarding the number and influence of publications. Research data show consistent results among leading countries, institutions, investments, and productive authors.

### 4.2 Research focuses

As shown in Figure 7 and Figure 8, the multiple roles of SEs in cancers, including cell identity, gene transcription, expression, and inhibition, as well as the relationship between SEs and TFs, and the selective inhibition of SEs, have received a lot of attention, suggesting that they are hot issues for research. SEs significantly drive the transcription of targeted genes and control cell identity by combining with a large number of tissue-specific TFs in cells, such as OCT4, SOX2, and Nanog (Whyte et al., 2013). DNA hypomethylation is related to the initiation and development of early tumors. And it is believed that local changes in TF binding affect the SE DNA methylation profile, thereby influencing target gene expression (Heyn et al., 2016). Thus, the keywords with high co-occurrence frequency included “super enhancer,” “cancer,” “expression,” “cell identity,” “transcription factor,” and “DNA methylation”.

The shift in research hotspots and the top 25 keywords with the most citations can be found in Figure 8 and Figure 9. The research focus was initially on the structure and function of SEs. BRD4 and Mediator complex subunit 1 (MED1) were first found to co-occupy SEs. BRD4, MED1, and P-TEFb are jointly involved in the transcriptional elongation of RNA Pol II (Lovén et al., 2013). Treatment with the BET-bromodomain inhibitor in tumor cells markedly reduced BRD4, Mediator, and P-TEFb levels at SEs and consequent transcription elongation defects that preferentially impacted genes with SEs (Lovén et al., 2013). Thus, the popular keywords included “P-TEFb” (2013–2015), “bromodomain protein BRD4” (2013–2016), “bet bromodomain inhibition” (2013–2017), and “bet bromodomain” (2013–2014).

SEs are associated with key oncogenes in cancers. Many oncogenes regulated by SEs have been identified by high-throughput sequencing technology, including *MYC*, composed of three paralogous genes *C MYC*, *N MYC,* and *L MYC* (Bahr et al., 2018). *Myc* dysregulation can be seen in 70% of human cancers (Dang, 2012). Thus “C MYC” is a keyword with a long duration of citation burst (2013–2015). With the further study of SEs, the mechanism of participating in the genesis and development of various tumors has been gradually revealed. Leukemia is the most studied cancer type, including acute myelogenous leukemia (2014–2017) and acute lymphoblastic leukemia (2014–2017). In human acute myeloid leukaemia, cell fate and phenotype, such as stem cell to terminal differentiation cell type, can be achieved by se regulating the expression of Myc (Bahr et al., 2018). In chronic myelogenous leukemia, the function maintenance of leukemia stem cells is highly dependent on the SE-driven gene transcription (Zhou et al., 2021).

With the clarification of the SEs’ function and the identification of super enhancer-related oncogenes in cancer, the inhibition of specific SE formation and SE-dependent oncogene transcription activation is considered a new strategy for novel therapeutic interventions in cancer (Lovén et al., 2013). Cancer cells can take advantage of SE-driven transcriptional dysregulation and become highly dependent on transcription to maintain their oncogenic state, termed transcriptional addiction, representing therapeutic vulnerabilities for targeting cancer cells (Sengupta and George, 2017). Small molecule inhibitors targeting critical components of SE complexes can eliminate oncogene addiction and thus interfere with tumor progression (Zheng et al., 2020). In addition to BRD4, CDK7 is another promising target for SEs (Lovén et al., 2013; Sava et al., 2020). Therefore, “selective inhibition” (2014–2016) and “covalent CDK7 inhibitor” (2017–2018) are the keywords with the highest citation strength. The anti-cancer effect of the covalent CDK7 inhibitor has been demonstrated in a variety of tumors, including leukemia (Zhou et al., 2021), bladder cancer (Liu et al., 2022), glioblastoma (Meng et al., 2018), oesophageal squamous cell carcinoma (Hu et al., 2019), and osteosarcoma (Taniue and Akimitsu, 2022). Mechanistically, the CDK7 inhibitor significantly disrupts SE-associated gene transcription by inhibiting CDK7 activity and reducing RNA pol II CTD phosphorylation (Zhang et al., 2020).

The aberrant super-enhancer landscape is established by master TFs and mediators at key cell identity genes (Whyte et al., 2013). A set of master TFs co-occupancy at their own SEs and each other’s, forming a core regulatory circuitry (CRC) to control the transcriptional programs (Boyko and Surewicz, 2022). These master TFs form a SEs-based CRC that determines the status of specific cell types in malignant cancer cells. The CRC model was first established in human embryonic stem cells. The transcription factors OCT4, SOX2, and NANOG collaborate to form an interconnected regulatory loop (43). A comprehensive and interactive database (dbCoRC, http://dbcorc.cam-su.org) of CRC models was established in 2018, which is inferred from the mapping of SEs and prediction of TF binding sites (44). Based on the SE modeling and TF assessments, CRCs and master TFs have been defined in chronic lymphocytic leukemia (45), lung adenocarcinoma (46), and esophageal squamous cell carcinoma (47, 48). Moreover, perturbation of transcriptional circuitry with small molecule inhibitors suppresses the expression of tumor-specific CRC TFs, exhibiting a prominent anti-cancer effect in multiple cancer types (45, 47, 49). Due to the specificity of CRC regulatory patterns, the interaction between SEs and TF may need to be focused on when developing therapeutic strategies. CRC-guided tumorigenesis mechanisms and rational therapeutic strategies are promising fields for future research.

### 4.3 Research frontiers and prospects

As we discussed above, SEs and regulated genes play important roles in the biological process of cancer cells, and therapy targeting SEs is a promising cancer treatment strategy. Recent research focuses on developing small molecule inhibitors targeting SE components, especially BRD4 and CDK7 inhibitors (Xiang-Ping Li et al., 2023). Pre-clinical studies and undergoing clinical trials showed significant antitumor efficacy of these inhibitors. However, limited clinical applications of inhibitors are available because of significant toxicity and adverse events (Xiang-Ping Li et al., 2023). It has been demonstrated in a study that the BRD4/LSD1/NuRD complex is destroyed by the BRD4 inhibitor JQ1 when used for a long period, leading to resistance to JQ1 and a broad spectrum of antitumor compounds (Liu et al., 2022). SY-1365 is a selective CDK7 inhibitor that entered the phase I clinical trial in 2017, and preliminary findings showed average efficacy (Hu et al., 2019). Therefore, it is necessary to develop further more efficient inhibitors targeting SEs to be truly applied to cancer treatment in the future. Previous studies on anti-cancer drugs have focused on cancer genomics, designing drugs for abnormal protein activation caused by mutations. However, this treatment strategy has limitations due to the tumor heterogeneity and the low mutation frequency of some genes (53). Recently, with the development of epigenomics, drug design based on epigenetic mechanisms has been of great significance (54). In the future, targeting aberrant DNA methylation, chromatin states, and histone modifications are promising directions for new drug design. Considering the key oncogenes and tumor biological processes regulated by SE in new drug design may be a new direction.

The CRISPR/Cas9 knockout system can be applied to target core regions of SEs to suppress the expression of SE-driven oncogenes. Disruption of the SE region of the RUNX1 gene in AML was demonstrated to promote cell apoptosis and alter the survival of mice with AML (55). However, the CRISPR/Cas9 system may cleave the non-targeting sites in cells, causing off-target effects, which limits its clinical application (56). Future studies should focus on effectively addressing off-target effects and reducing clinical risks. The newly improved CRISPR affinity purification *in situ* of regulatory elements (CAPTURE) technology is based on the CRISPR elements. It adds biotin ligase BirA, facilitating the systematic dissection of SE components and SE function (57). In conclusion, editing targeted SEs using CRISPR/Cas9 technology can explore and verify the significant role of SEs in tumor progression.

Recently, the dynamic activation of SEs in tumors has received increasing attention. In eukaryotic cells, biomolecular condensates coordinate specific molecules and biological reactions, and the key mechanism of their formation is liquid-liquid phase separation driven by multivalent and weak macromolecular interactions (Taniue and Akimitsu, 2022). Dysregulation of LLPS leads to many human diseases, including neurodegeneration and cancer (Boyko and Surewicz, 2022; Taniue and Akimitsu, 2022). Once the concept of phase separation was proposed, it attracted extensive attention from scholars at home and abroad. Science ranked phase separation as the runner-up in its 2018 breakthrough journal (60). Master TFs and the Mediator coactivator are reported to form phase-separated condensates at SEs, which compartmentalize the transcription apparatus and concentrate them on the key cell identity genes (Sabari and Dall'Agnese, 2018). Signaling factors of WNT, TGF-β, and JAK/STAT pathways enter and concentrate into the phase-separated condensates at SEs of cell identity genes (61). A recent study showed that the CRC components HOXB8 and FOSL1 can form phase-separated condensates at SE loci. H3K27 demethylase inhibitor can destroy this CRC phase separation, thus leading to metastasis inhibition and re-sensitivity to chemotherapy drugs, which provides a new strategy for treating metastatic and chemoresistant osteosarcoma (62). Interestingly, phase-separated condensates can also concentrate anti-cancer drugs *in vitro* and tumor cells, such as cisplatin and tamoxifen, contributing to drug pharmacodynamics (63). Given the ability of drugs to concentrate on specific condensates, it should be reasonable to design small molecules that can target specific condensates when designing drugs.

However, there is a huge controversy about whether phase separation can occur in cells and whether it is important to cell biological processes. Phase separation is an unrecognized mechanism for arranging cell contents and aggregating molecules that trigger key cell events. However, the opponents believe that the research method on phase separation is unreliable. The current research results cannot prove that phase separation occurs *in vivo*. Moreover, in vitro reconstruction experiments, existing optical microscopy techniques are difficult to deeply observe the fine structure of phase separation. The development of new technologies is required to analyze the fine structure of phase separation. Meanwhile, how phase-separated condensates contribute to tumorigenesis remains unclear (60). The current studies mainly focus on the role of biomolecular condensates in cancer, lacking the intrinsic mechanism for the dynamic process of phase separation. Therefore, new research tools are urgently needed to understand and better analyze the fine structure of phase separation in cells and the role of phase separation in tumor progression.

## 5 Conclusion

Since 2013, research on the relationship between SE and cancer has grown rapidly. The United States continues to play a leading role in this field, as the top literature numbers, affiliations, funding agencies, and authors were all from the United States, followed by China and European countries. Countries have carried out active and fruitful cooperation for SE and tumor-related research. The role of SEs in cell identity, gene transcription, expression, and inhibition, as well as the relationship between SEs and TFs, and the selective inhibition of SEs, have received much attention, suggesting that they are hot issues for research.

## Data Availability

The original contributions presented in the study are included in the article/supplementary material, further inquiries can be directed to the corresponding author.
