# Corrigendum: Bibliometric analysis of evolutionary trends and hotspots of super-enhancers in cancer

**DOI:** 10.3389/fphar.2023.1285522

**Published:** 2023-09-05

**Authors:** Zhen-Chu Tang, Qiang Qu, Xin-Qi Teng, Hai-Hui Zhuang, Wei-Xin Xu, Jian Qu

**Affiliations:** ^1^ Department of Neurology, The Second Xiangya Hospital of Central South University, Changsha, China; ^2^ Hunan Key Laboratory of Tumor Models and Individualized Medicine, The Second Xiangya Hospital of Central South University, Changsha, China; ^3^ Department of Pharmacy, Xiangya Hospital, Central South University, Changsha, China; ^4^ National Clinical Research Center for Geriatric Disorders, Xiangya Hospital, Central South University, Changsha, China; ^5^ Institute of Hospital Management, Central South University, Changsha, China; ^6^ Hunan Key Laboratory of the Research and Development of Novel Pharmaceutical Preparations, Changsha Medical University, Changsha, China; ^7^ Department of Pharmacy, The Second Xiangya Hospital, Institute of Clinical Pharmacy, Central South University, Changsha, China

**Keywords:** super-enhancer, bibliometric analysis, cancer, hot spots, trends

In the published article, there was an error regarding the **affiliation** for author Jian Qu. Instead of “Jian Qu^6,7*^” it should be “Jian Qu^7^*”. Jian Qu’s correct affiliation is “^7^Department of Pharmacy, The Second Xiangya Hospital, Institute of Clinical Pharmacy, Central South University, Changsha, China” and the following affiliation is incorrect and should be removed “^6^Hunan Key Laboratory of the Research and Development of Novel Pharmaceutical Preparations, Changsha Medical University, Changsha, China”.

The authors apologize for this error and state that this does not change the scientific conclusions of the article in any way. The original article has been updated.

